# Tai Chi practitioners have lower fall risks under dual-task conditions during stair descending

**DOI:** 10.1371/journal.pone.0246292

**Published:** 2021-02-04

**Authors:** Yang Li, Qipeng Song, Li Li, Wei Sun, Cui Zhang

**Affiliations:** 1 Department of Physical Education, Nanjing Forestry University, Nanjing, Jiangsu, China; 2 College of Sports and Health, Shandong Sport University, Jinan, Shandong, China; 3 Department of Health Sciences and Kinesiology, Georgia Southern University, Statesboro, Georgia, United States of America; 4 Lab of Biomechanics, Shandong Institute of Sport Science, Jinan, Shandong, China; The Wingate College of Physical Education and Sports Sciences at the Wingate Institute, IL, ISRAEL

## Abstract

Stairs are among the most hazardous locations, and stair descending contributes to a high risk of falls among the elderly under dual-task (DT) conditions. The purpose of this study was to determine whether the practitioners of Tai Chi (TC), one type of mind-body exercise, have lower fall risks under DT conditions during stair descending, compared with their no-exercise (NE) counterparts. Fifteen TC practitioners with at least 10 years of experience in TC and fifteen NE participants were recruited in this study. They were asked to descend a six-step staircase under single-task (ST) and DT conditions. An eight-camera motion analysis system and two force plates were used for data collection. Results showed group by DT interactions in walking velocity (p = 0.016) and center of mass–center of pressure inclination angle (COM–COP IA) in the anteroposterior directions (p = 0.026). Group effects observed with foot clearance (p = 0.031), trunk (p = 0.041) and head (p = 0.002) tilt angles, and COM–COP IA in the mediolateral (p = 0.006) directions. Significant DT effects only detected in foot clearance (p = 0.004). Although both groups of participants adopted a more cautious gait strategy under the dual-task condition, the TC practitioners were less influenced by the DT paradigm than their NE counterparts. Our observations indicated that TC practitioners have lower fall risks under DT conditions during stair descending.

## Introduction

Taking the stairs is common in daily life; 10% of home accidents [1], 14% of occupational injuries related to work surfaces [2], and 26% of self-reported falls occur on stairs [3]. The continuously increasing number of the stair-related injuries [4] has made stairs one of the most hazardous locations for fall accidents [5] and the leading cause of accidental death for the elderly [3]. Stair descending accounts for 75% of falls among the elderly in communities [6]. Compared with level walking, stair descending demands high lower limb joint range of motion [7] and muscle strength [8], imposes a significant challenge to postural control in the elderly [9].

Some variables were related to falls during stair negotiation, like walking velocity, foot clearance, trunk and head tilt angles, and center of mass-center of pressure inclination angle (COM-COP IA). The component responsible for the lower margin of stability in the elderly was the higher velocity of the COM leading to a more anterior position of the extrapolated COM in relation to the edge of the step. A higher walking velocity increases the difficulty of postural control in the anterior direction among older adults [8]. Catching the foot on the stair edge and foot misplacement on the stair are the main causes of trips during stair descending [10]. Trips can be avoided by maintaining foot clearance greater than zero on any given step [11]. In the event of an overstep, increased forward upper body tilt angles (trunk or head tilt angles) in the sagittal plane put forward higher requirements on postural control during stair descending. This is particularly important for older adults, in light of the significant age-related changes in neuromuscular function which would challenge one’s ability to counter the forward momentum of the upper body when it is placed further forward due to trunk flexion [12]. Measurement of the body’s COM motion relative to the COP is essential for quantifying postural control because their relative positions are directly related to the COM’s gravitational moment, which should be counterbalanced to prevent the whole body from falling [13]. The direction of the COM-COP vector can be described by the angle formed by the vertical and the projection of the vector onto the sagittal and frontal planes, termed COM-COP IA [14].

In a dual-task (DT) paradigm, the introduction of a concurrent task (mostly a cognitive task) during a primary task (mostly a physical task) leads to possible competition among the attentional resources available [15]. The risk of falls increases while performing a concurrent cognitive DT, such as talking, calculating, and reasoning [9], by increasing stride time variability [16], decreasing the extremity joint range of motion [17], and decreasing foot clearance during stair waking [18]. Among the elderly, postural control impairment under DT conditions is a common occurrence [19]. Impaired postural control under DT conditions predicts adverse outcomes, such as falls [20]. Previous research indicated that physical tasks’ difficulty influences DT performance [21], and this effect might be higher in older adults than in young adults [22].

Exercise has been proven effective in preventing falls and decreasing the risk of falls, and one of its most popular forms is Tai Chi (TC) [23]. TC, often described as “moving meditation,” encompasses physical and mental elements [24]; integrates training in balance, flexibility, and neuromuscular coordination with several cognitive components, including heightened body goal-oriented training; and may result in benefits to reducing fall risks and improving cognitive performance [16]. However, the potential of TC as a mind-body exercise to reduce fall risks under DT conditions has not received considerable attention.

The ability to maintain balance under DT conditions is dependent on the efficiency of attentional resources [17], which controls physical and cognitive task performances. Interference between two tasks suggests that shared attentional resources may involve the regulation of postural control and cognitive performance. Research utilizing the DT paradigm can provide new insights into the interactions between them [22]. Evidence suggests that multimodal training that integrates physical and cognitive elements may be more effective than any one-element intervention [25]. TC can improve DT performance as a multimodal mind-body exercise that incorporates physical, cognitive, social, and meditative components. However, a limited number of existing studies have provided the scientific basis and therapeutic impetus to explore further the cognitive and physical benefits of TC [26]. At present, no drug therapy has been proven to delay preclinical cognitive deterioration [27]. If certain exercises are confirmed to work, then they would benefit the elderly population. We hypothesized that TC practitioners have lower fall risks under DT conditions during stair descending, compared with their NE counterparts.

## Materials and methods

### Participants

A priori power analysis (G*Power Version 3.1) indicates that a minimum of 9 participants are needed in each group to obtain the alpha level of 0.05 and the beta level of 0.80 based on a previous report that compared the COM-COP IA between DT and ST conditions during stair descending [28]. In the current study, 30 elderly participants were recruited after distributing flyers and providing presentations in a local community in Jinan, China. Of the 25 participants, 13 were amateur TC practitioners, with TC experience of 19.7±6.4 years, and 12 were no-exercise (NE) practitioners (See Table 1). All participants were right foot dominant; that is, they prefer the right leg in kicking a football. The inclusion criteria were age≥65 years, regularly practiced TC for at least 10 years for half an hour per day three times a week for TC practitioners, and three months of no specific exercise (total exercise time should be less than 1 h per week) for NE participants. The exclusion criteria were inability to follow instructions, unstable heart conditions, joint replacements in the lower extremities, arthritis, diabetes, visual deficits, vestibular deficits, falls in the last 3 years, or any type of neuromuscular problems that could prevent the participants from meeting the project requirements. Independent t-tests showed no significant differences in age, height, and body mass between groups. All the practitioners signed approved informed consent forms prior to participation. The use of human participants in this study was approved by the Internal Review Board of Shandong Sport University (2017SC-014) and was in accordance with the Declaration of Helsinki.

**Table 1:** Demographic characteristics of practitioners in TC and control groups.

	TC group	NE group	Between-Group Comparison
	Mean±S.D.	Mean±S.D.	*p*-Value
Individuals (n)	13	12	----
Age (years)	70.3±3.2	71.8±4.4	*p*>0.05
Height (cm)	163.6±8.2	161.4±6.9	*p*>0.05
Body Mass (kg)	64.6±8.6	62.1±9.1	*p*>0.05
Gender (F/M)	8/5	7/5	----
TC experience (years)	19.7±6.4	----	----

TC: Tai Chi.

NE: No exercise.

S.D.: Standard Deviation.

### Lab setting and testing protocol

A cast-iron staircase with six steps was constructed for data collection. The step dimensions were 17.0 cm (riser) × 29 cm (tread). Two force platforms (KISTLER, 9287BA and 9281CA, Switzerland) were embedded in the third and fourth steps of the staircase to collect ground reaction force data at a sample rate of 1,000 Hz [29]. The stair descending test was recorded by an eight-camera motion analysis system (Vicon, Oxford Metrics Ltd., England) at 100 Hz. The Vicon system synchronized the force platforms and motion analysis system. The participants were asked to ascend the staircase in a step-over-step manner under two conditions. Condition 1 (ST) included stair descending only, and condition 2 (DT) included stair descending while subtracting the serial sevens from a three-digit number [18]. Before the tests, the participants were allowed to warm up by walking for 400 m at a self-selected velocity. They were then asked to ascend and descend the staircase several times to familiarize themselves. During the test, each participant was asked to perform five successful stair descending trials. A successful stair descending trial was defined as a trial in which the participant continuously ascended from the top to the end of the staircase. The participants had 1 min to rest after each trial. Each participant used the same pair of shoes for all testing sessions.

### Variables

Walking velocity was calculated as the displacement of the COM divided by step time. Foot clearance was calculated as the vertical distance between the heel marker and stair edge when the heel marker was above the edge [30]. Head/trunk tilt angle was defined as the maximal angle between the head/trunk longitudinal axis and the horizontal plane projected to the sagittal plane [29]; a positive angle indicated leaning forward. COM-COP IA was determined as the instantaneous orientation of the line connecting the COM and COP with respect to a vertical line through the COP [31]. The COP position was calculated using forces measured by the force plate. The anterior (+)/posterior (-) positions of the COM and COP are described parallel to the direction of progression. The medial (left)/lateral (right) positions of COM and COP are described relative to the line of progression [32]. IA in anterior/posterior and medial/lateral was calculated correspondingly (see below). In this study, we examined the maximum IA during a single-support phase, an unstable period in which one can easily fall [33].

The anterior/posterior COM-COP IA (α) and the medial/lateral COM-COP IA (β) were calculated as follows [34]:

$$\vec{\text{t}}=\frac{{\vec{\text{P}}}_{\text{COM}-\text{COP}}\times {\vec{\text{P}}}_{\text{vertical}}}{{\vec{\text{P}}}_{\text{COM}-\text{COP}}}$$ (1)

$$\alpha ={\text{sin}}^{-1}({\text{t}}_{\text{x}})$$ (2)

$$\beta ={\text{sin}}^{-1}({\text{t}}_{\text{y}})$$ (3)

where $\overset{⃑}{P}$COM–COP is the vector pointing from COP to COM, and $\overset{⃑}{P}$vertical is the unit vector of the vertical axis of the global coordinate system.

### Data reduction

All variables were obtained from the Vicon system and then exported to Microsoft Excel 2016 for further calculation. A 13-segment whole-body model, from which body COM was obtained using segment anthropometric data, was established [35]. Kinematics data were obtained on the stride beginning with foot contact with the fourth step of the staircase and ending with the same foot contact with the second step. Kinetics data were obtained on the right foot contact with the third or fourth step. All kinetics data were normalized by body weight. The kinematic data were down-sampled to match the sampling rate with the kinetic data. The kinematics and kinetics data included a fourth-order low-pass Butterworth filter with cutoff frequencies of 7 Hz [36] and 50 Hz [37], respectively.

### Statistical analysis

The normality of all outcome variables was tested using the Shapiro–Wilk test. The mean and standard deviations for the variables above were subjected to descriptive analysis. A two-way ANOVA with repeated measures was used to determine the effects of the group and the DT paradigm on each outcome variable. Bonferroni-adjusted post hoc analysis was conducted when the significant group–DT interactions were detected. Partial eta squared (*η*2_p_) was used to represent the effect size of the two-way ANOVA’s main effects and interactions. The thresholds for *η*2_p_ are as follows: 0.01–0.06, small; 0.06–0.14, moderate; and >0.14, large [38]. Cohen’s *d* was used to represent the effect size of the post hoc comparison. The thresholds for Cohen’s *d* are as follows: <0.20, trivial; 0.21–0.50, small; 0.51–0.80, medium; >0.81, large [39].

## Results

**Fig 1:**
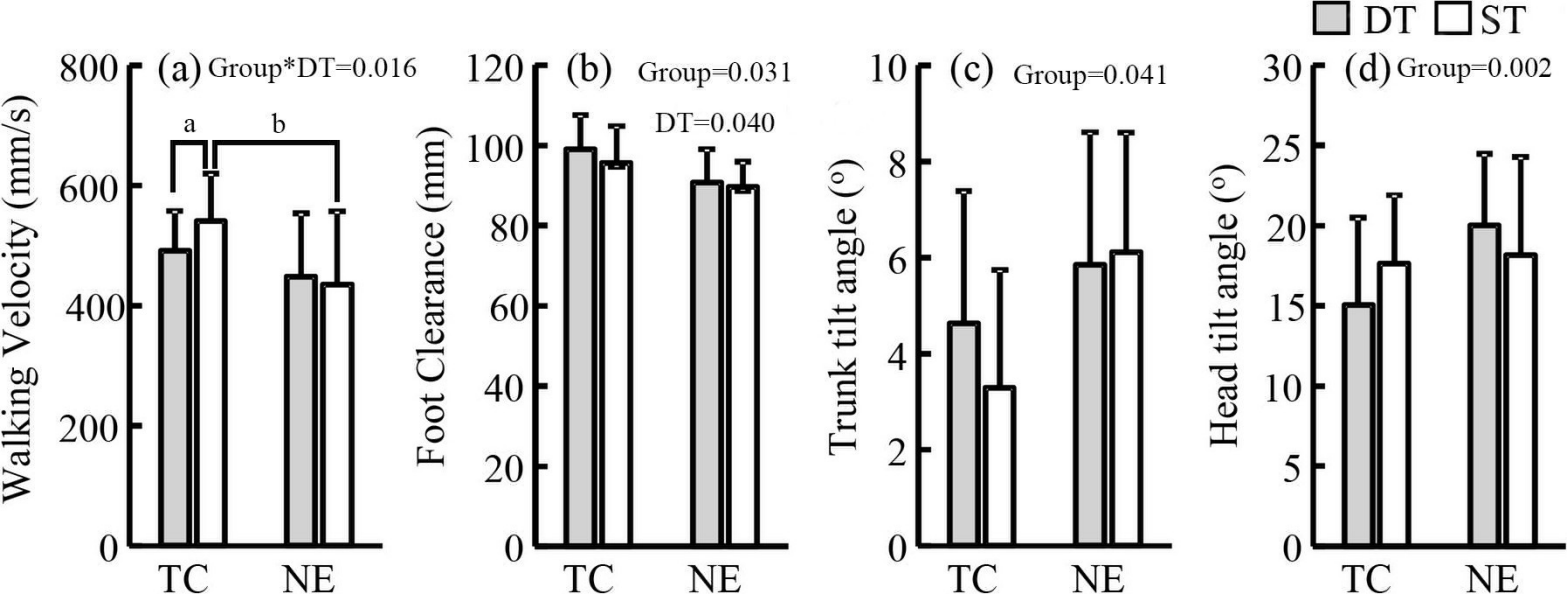
Kinematic variables during stair decent. TC: Tai Chi, NE: no-exercise, DT: dual task, and ST: single task. a: Significant difference between the DT and ST conditions among the TC practitioners. b: Significant difference between TC and NE participants under the ST conditions.

The Shapiro–Wilk test indicated that all the dependent variables were normally distributed (p>0.05). Fig 1 presents the outcomes of the kinematic variables during stair descending. Significant group–DT interactions were detected in walking velocity (p = 0.016, *η*^2^_p_ = 0.226). Under the DT and ST conditions, the TC practitioners had a larger foot clearance (p = 0.031, *η*^2^_p_ = 0.187), a smaller trunk (p = 0.041, *η*^2^_p_ = 0.169) and head (p = 0.002, *η*^2^_p_ = 0.340) tilt angles than the NE practitioners. The TC practitioners had a higher walking velocity (p = 0.016, Cohen’s *d =* 1.027) under the ST conditions than the NE practitioners under the DT condition. Under the DT condition, the TC and NE practitioners increased their foot clearance (p = 0.040, *η*^2^_p_ = 0.171) relative to those under the ST conditions. Under the DT conditions, the TC practitioners decreased their walking velocity (p = 0.007, Cohen’s *d =* 1.730) relative to those under the ST condition.

**Fig 2:**
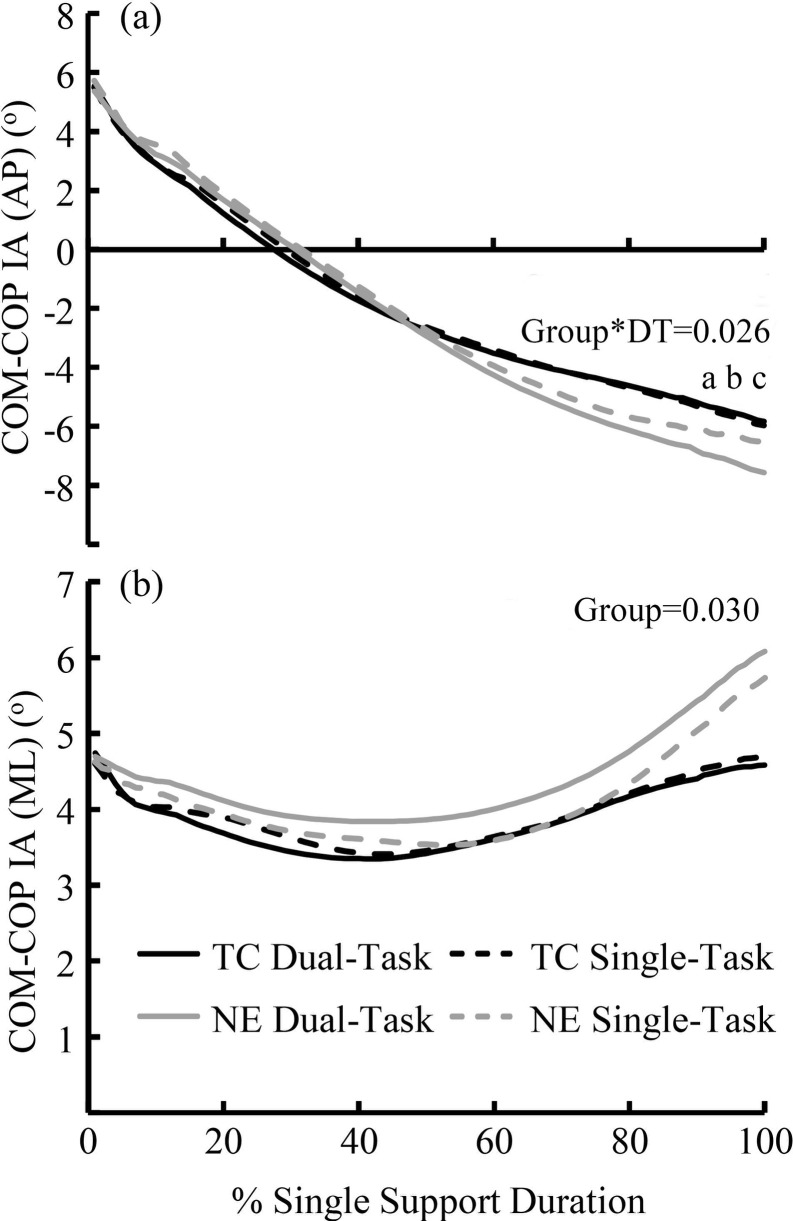
Center of mass–center of pressure inclination angle (COM–COP IA). TC: Tai Chi group, NE: no-exercise group. COM-COP AI: Center of Mass-Center of Pressure Inclination Angle. AP: anteroposterior direction, ML: mediolateral direction. a: Significant difference between the DT and ST conditions among the NE practitioners. b: Significant difference between the TC and NE practitioners under the DT conditions. c: Significant difference between the TC and NE practitioners under the ST conditions.

Fig 2 presents the COM–COP IA in the anteroposterior (a) and mediolateral (b) directions. In the anteroposterior directions, a significant group–DT interaction was detected (p = 0.026, *η*^2^_p =_ 0.198). Under the DT conditions, the NE practitioners showed an increased COM–COP IA relative to that under the ST condition (p = 0.001, Cohen’s *d =* 1.346). TC practitioners had a smaller COM-COP IA under the DT (p = 0.005, Cohen’s *d* = 1.166) and ST (p<0.001, Cohen’s *d* = 2.163) conditions. In the mediolateral directions, the TC practitioners had a smaller COM-COP IA than the NE practitioners (p = 0.005, *η*^2^_p =_ 0.297) under the DT and ST conditions.

## Discussion

We aimed to investigate the effects of TC exercise on reducing fall risks of the elderly during stair descending under DT conditions. The results support our hypothesis that TC practitioners have lower fall risks under DT conditions during stair descending, compared with their NE counterparts.

Our outcomes indicated that the lower fall risks under the DT condition among the TC practitioners might be related to some of their unique gait strategies, such as decreased walking velocity. Lower walking velocity decreases the difficulty in controlling stability during stair descending [40]. Long-term TC practitioners have been reported to decrease their walking velocity when the ambient brightness decreases [40]; our outcome agrees with their viewpoint that TC practitioners attempt to stabilize themselves by decreasing their gait velocity under challenging conditions.

Compared with the NE practitioners, the TC practitioners had higher foot clearance under the DT and ST conditions during stair descending. Falls generally occur after tripping on a stair edge [41]. A high foot clearance decreases the risk of catching a heel on the stair edge during the swing phase, thereby decreasing the likelihood of a trip [10]. In this work, the TC and NE practitioners took a more cautious gait strategy under the DT condition than under the ST condition, as represented by the increased foot clearance. A previous study pointed out that TC practitioners do not increase their foot clearance under challenging conditions, i.g., low illumination condition [40]. The difference between our study and the previous one may be attributed to the different responses of the elderly under the DT and low illumination conditions.

During TC, practitioners generally raise their legs and stay still for a relatively long period. The regular practice of these movements may increase foot clearance during stair descending. In the present study, the TC practitioners had lower head and trunk tilt angles than the NE practitioners. Excessive head and trunk tilt angles would move the COM forward, put forward higher requirements on postural control in the anterior direction during stair descending. Practitioners generally keep their trunks erect, and they control their posture with low limb movements during TC exercise. These movements in TC exercise may be applied to stair descending when TC practitioners held their heads and kept their trunks erect under DT and ST conditions.

COP through the feet and the COM of the body are commonly used to reflect postural control. Researchers have proposed that the COM and COP’s collaborative interpretation can provide a better assessment than examining COM or COP separately [42]. COM-COP IA, formed by the vertical line and the projection of the vector onto the sagittal and frontal planes [14], was proposed to provide a more comprehensive assessment than the examination of COM or COP [43], because COM or COP measures may not necessarily indicate instability [43], and COM-COP separation, was not normalized to leg length or body height [44]. When one lifts the foot to the desired direction, postural stability is challenged because the base of support is drastically reduced. If the COM is not repositioned above the new base of support, a gap between the COM and COP would be created, and the whole body would fall towards the swing leg side [45]. Greater IA leads to an increase in moment arm of force, which further increases inertia and momentum of the body segments for balance recovery [43]. The greater the IA, the further the COM diverges from the COP, and the more difficult it is to bring the COM above the COP [34]. If the COM and COP become separated extensively and consequently prevent the lower-limb joint moments from supporting upright posture, falls can occur [33]. Extensive research has proved that TC practitioners have greater postural stability than NE practitioners under ST conditions [46]; The current study fills the gap by demonstrating that TC practitioners have lower fall risks under DT conditions.

The increase in COM–COP IA in the anteroposterior directions among the NE practitioners indicated that their postural stability decreased under the DT condition. By contrast, the COM-COP IA remained unchanged among the TC practitioners. The decreased postural stability among the NE practitioners could be explained by a neuroimaging study, which suggested that gait and cognitive tasks may share a network of brain regions in the frontal and parietal cortex, often referred to as the frontoparietal executive control network [47]. Gait is not automatic; it requires attentional resources from the high brain center [18]. DT-related gait changes can result from the interference caused by a competition between the attention demands under the DT condition. Combined interventions, such as mind-body exercise in TC, have been confirmed to improve executive function [16], which refers to cognitive processes that orchestrate goal-directed activities and allocate attentional resources in competing tasks. Improved executive function can coordinate the competition between the attention demands by physical and cognitive tasks to reduce fall risks under DT conditions.

Postural control is related to the complex interactions between peripheral sensory input and motor processes modulated by CNS [48]. Proprioception and cutaneous sensitivity are primary components of the somatosensory system [49], which contributes 60%–70% for postural control, while visual and vestibular systems contribute to the rest [50, 51]. It has been documented that practicing Tai Chi could enhance both the proprioception [52] and the cutaneous sensitivity [53] among older adults. Furthermore, one of our previous studies indicated that Tai Chi practitioners had a better white matter integrity in the splenium of corpus callosum than their controllers, and inferred that the Tai Chi induced microstructure changes in CNS tissue are linked with memory and cognition improvements [54]. TC practitioners may take advantage of their CNS function and sensory input systems to “feel” the landing process and maintain postural stability during stair descending.

This study has several limitations. Firstly, the cognitive task used in this dual-task study has employed verbal responses. However, respiration changes during speech production are known to produce changes in postural control [55]. Secondly, the cognitive performance during the dual-task was not assessed, and therefore the trade-off between cognitive and balance tasks cannot be determined. Thirdly, the visual system condition was not well controlled in our study, which may alter the participants’ motor performance [56]. Finally, only the physical–cognitive combination under the TC exercise can be verified to fall risks under the DT condition. Carrying out a study containing single physical and cognitive exercises would help verify whether a combination exercise has a greater effect than a single physical exercise on reducing fall risks.

Future studies should first overcome these limitations. We recommend further studies containing physical (such as walking or jogging), cognition (such as meditation) and body-cognitive combination (such as Tai Chi or yoga) exercises should be conducted, to identify the benefits of each category of exercise style to reducing fall risks, and further provide objective rehabilitation programmes for the cognitively impaired population.

## Conclusions

Although both groups of participants adopted a more cautious gait strategy under the dual-task condition, the TC practitioners were less influenced by the DT paradigm than their NE counterparts. Our observations indicated that TC practitioners have lower fall risks under DT conditions during stair descending.

## Supporting information

S1 Raw data (XLSX)
